# High-Quality Genome Reconstruction of Candida albicans CHN1 Using Nanopore and Illumina Sequencing and Hybrid Assembly

**DOI:** 10.1128/MRA.00299-21

**Published:** 2021-06-10

**Authors:** Shipra Garg, Piyush Ranjan, John R. Erb-Downward, Gary B. Huffnagle

**Affiliations:** a Department of Molecular, Cellular, and Developmental Biology, University of Michigan, Ann Arbor, Michigan, USA; b Division of Pulmonary and Critical Care Medicine, University of Michigan, Ann Arbor, Michigan, USA; c Department of Microbiology and Immunology, University of Michigan, Ann Arbor, Michigan, USA; d Mary H. Weiser Food Allergy Center, University of Michigan, Ann Arbor, Michigan, USA; University of California, Riverside

## Abstract

We report an improved, nearly closed, high-quality draft genome reconstruction of the Candida albicans CHN1 strain (ATCC MYA-4779), a human isolate, using Illumina and Nanopore sequencing. Covering six complete and two partial nuclear chromosomes along with a partial mitochondrial genome, this assembly is 14,787,852 bases in size, with 5,935 genes.

## ANNOUNCEMENT

The Candida albicans CHN1 strain is a human isolate that has been shown to stably colonize cefoperazone-pretreated mice (1–3). DNA sequencing of this isolate was performed using the long-read Nanopore MinION and high-accuracy Illumina MiSeq platforms, with a hybrid assembly being used for genome reconstruction. Yeast cells were grown in Sabouraud dextrose broth (Difco, Detroit, MI) at 37°C. The Qiagen DNeasy blood and tissue kit was used to isolate DNA for Illumina sequencing. Library preparation using the PrepX DNA library kit (number 640101; TaKaRa) was followed by bead size selection for inserts of 220 bp. Sequencing on the MiSeq platform was performed with 500 cycles at the University of Michigan DNA Sequencing Core. A total of 6,941,089 raw read pairs (2 × 251 bp) were subjected to adapter trimming using TrimGalore v0.5.0 (https://github.com/FelixKrueger/TrimGalore) and Cutadapt v1.18 (4) with a minimum Phred score of 30. The resulting 6,820,861 paired-end reads were then merged together using FLASH v1.2.11 (5) with a minimum overlap of 25 nucleotides, leading to 6,634,784 single-end reads. High-molecular-weight DNA for Nanopore sequencing was isolated using the Zymolyase-based extraction protocol recommended by Oxford Nanopore Technologies (ONT) for yeast DNA, with modifications from the Qiagen DNeasy blood and tissue kit. Library preparation was performed using the rapid sequencing kit SQK-RAD004 from ONT, and no size selection or shearing was applied. Nanopore sequencing was performed locally on the MinION platform. A total of 485,750 raw reads, with an estimated *N*_50_ of 23,280 bases, were obtained after base calling with Guppy v3.2.9. Reads were trimmed and filtered to remove adapter contamination using SNIKT v0.1.1 (https://github.com/piyuranjan/SNIKT) and seqtk v1.3 (https://github.com/lh3/seqtk). NanoPlot v1.28.2 (6) was used for quality checking (380,747 single-end reads, with a read *N*_50_ of 20,290 bases).

*De novo* assembly of the Nanopore reads was performed using Flye v2.7 (7) with three iterations of error correction. The Illumina high-accuracy short reads were used to perform another independent error correction (polishing) of the contigs using BWA v0.7.17 (8) and Pilon v1.23 (9). The resulting assembly contained 23 contigs, with a largest contig length of 3,195,262 bases, a total throughput of 14,787,852 bases, an assembly *N*_50_ value of 1,642,426 bases, and a GC content of 33.62%, as assessed by QUAST v5.0.2 (10). BUSCO v4.0.6 (11) was used with the saccharomycetes_odb10 data set to estimate the genome completeness of the assembly as 98.1% (of 2,137 benchmarking universal single-copy ortholog [BUSCO] groups) with 35 missing core single-copy genes (CSCGs) from this lineage at the class level. As a comparison, C. albicans strain SC5314 (NCBI assembly number ASM18296v3 and RefSeq accession number GCF_000182965.3) has a completeness of 98.8% with 22 missing CSCGs, suggesting that some of these genes may no longer constitute the core C. albicans genome. The assembly contigs were then aligned with the strain SC5314 genome using MUMmer v4.0.0beta2 (12) and analyzed using Mauve v20150226 (13) for estimation of chromosomal completeness, organization, and synteny. Chromosomes 1 to 6 were recovered in full in this assembly. Chromosomes 7 and R were recovered in two and three long contigs, respectively. The mitochondrial genome was recovered in three contigs. Several small fragments that overlapped genomic regions in the SC5314 and CHN1 synteny comparisons were also retrieved. This genome is known to be diploid (14), and overlaps may represent sections of chromosomes with alternate alleles.

### Data availability.

This whole-genome shotgun project has been deposited in DDBJ/ENA/GenBank under the accession number JAFFGX000000000. The version described in this paper is version JAFFGX010000000. The C. albicans CHN1 GenBank Assembly accession number is GCA_017309835.1. The raw reads are available under accession numbers SRX9854709 (Illumina) and SRX9854710 (Nanopore) within BioProject PRJNA692229.
